# Supplementary material for: Extended longevity of termite kings and queens is accompanied by extranuclear localization of telomerase in somatic organs and caste‐specific expression of its isoforms
Source: Insect Sci. 2024 Jul 21;32(2):364–84. doi: 10.1111/1744-7917.13418 (PMC11976694; doi:10.1111/1744-7917.13418)
Supplement: Supplementary file 1 — Fig. S1 PCR detection of psTERT isoforms. [file INS-32-364-s001.pdf]

Table S1. List of primers used for qPCR-based expression profiling of *psTERT* isoforms

Primer name	Target region	Orientation	Sequence	Product length	Primer efficiency
psTERT-altTSS_A-F	TERT1	F	CGCTGTTACAGAGCTGTGGTC	182 bp	102.5 %
psTERT-altTSS_A-R		R	TCAGTGCTGACATTTGGCTTTA		
psTERT-TSS1-qPCR-F	TERT2	F	TCCAACGTGTAATTGTTCAGACA	148 bp	101.3 %
psTERT_EX2_R1		R	ACACAAGACTTGGTTGAAGATGGA		
psTERT-TES-qPCR-F2	TERT-B	F	AGATATTGCCCATTCCATGC	340 bp	105.7 %
psTERT-TES1-qPCR-R		R	TCTGTCTCTATATTTTCTTGGCGC		
psTERT-TES-qPCR-F2	TERT-A	F	AGATATTGCCCATTCCATGC	351 bp	99.9 %
psTERT-TES2-qPCR-R3		R	TACATCATCCAGCGAACAGC		
psGAPDH-F	GAPDH	F	GTCGCTTCAAGGGTGAAGTT	166 bp	98.3 %
psGAPDH-R		R	GATGCCTTTTCGATGGTTGT		

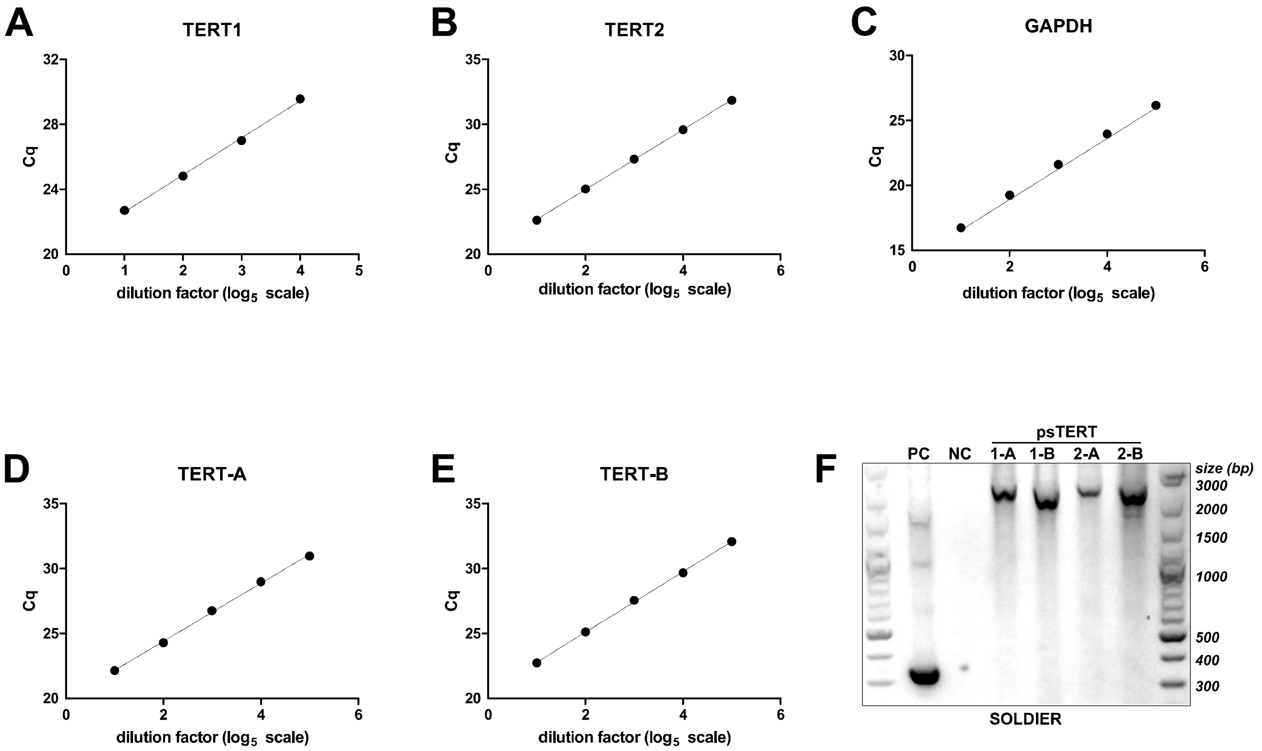

Figure S1. PCR detection of *psTERT* isoforms. **A-E**. Calibration curves of Cq values for cDNA serial dilution series used for calculation of qPCR primer efficiencies of *TERT1, TERT2, TERT-A, TERT-B* and *GAPDH* assays. **F**. PCR detection of all four splice variant combinations *TERT1-A, TERT1-B, TERT2-A* and *TERT2-B* in cDNA isolated from *P. simplex* soldiers. PC - positive control prepared using primers specific for splice variant *psTERT1*, NC - no template control.

Table S2. Expression of *psTERT* in RNA-seq data from various castes and tissues

Caste	Tissue	Sample ID	Total number of reads	TERT read counts	FPKM	FPKM mean	FPKM SD
mature neotenic gueen (>2 years old)	abdominal soma	MFB1	5,248,186	78	5.48	**5.64**	*0.48*
		MFB2	5,046,248	71	5.19		
		MFB3	7,122,462	122	6.32		
		MFB4	7,643,969	115	5.55		
mature neotenic gueen (>2 years old)	reproductive organs	MFG1	9,277,670	256	10.18	**12.58**	*2.16*
		MFG2	9,366,589	296	11.66		
		MFG3	7,322,612	302	15.22		
		MFG4	6,321,494	227	13.25		
mature neotenic gueen (>2 years old)	head	MFH1	5,551,562	160	10.63	**10.84**	*0.15*
		MFH2	7,986,297	238	11.00		
		MFH3	4,880,298	144	10.89		
		MFH4	8,680,936	255	10.84		
pseudergate (worker)	abdominal soma	WB1	7,966,238	130	6.02	**6.36**	*0.47*
		WB2	8,509,544	143	6.20		
		WB3	8,099,659	155	7.06		
		WB4	8,334,976	139	6.15		
pseudergate (worker)	head	WH3	7,108,756	235	12.20	**11.69**	*0.71*
		WH4	9,899,490	294	10.96		
		WH5	5,370,084	163	11.20		
		WH6	6,403,463	215	12.39		
young neotenic gueen (3< month old)	abdominal soma	YFB1	7,457,596	128	6.33	**7.50**	*1.57*
		YFB2	6,351,011	116	6.74		
		YFB3	7,457,854	198	9.80		
		YFB4	6,983,818	135	7.13		
young neotenic gueen (3< month old)	reproductive organs	YFG1	8,760,328	412	17.35	**15.32**	*2.24*
		YFG2	9,471,560	366	14.26		
		YFG3	6,377,926	294	17.01		
		YFG4	7,278,176	250	12.68		
young neotenic gueen (3< month old)	head	YFH1	8,560,172	154	6.64	**11.05**	*4.27*
		YFH2	9,626,068	296	11.35		
		YFH6	6,938,810	285	15.16		

Two-way ANOVA for abdominal soma and heads of workers, young queens and old queens

Source of Variation	% of total variation	F	P value
Interaction	0.38	0.08582	0.9182
Body part Factor	54.02	24.27	0.0001
Caste Factor	9.46	2.126	0.1500

t-test for gonads in young vs. mature queens

t	P value	df
1.496	0.1852	6

Table S3a. Test statistics relative to the results presented in Figure 2A–F

TERT-A (Figure 2A)

BROWN-FORSYTHE test				
Df:	6, 21			
F:	1.1762			
P value:	0.3561			
**ONE-WAY ANOVA**				
Df:	6, 21			
F:	67.6236			
P value:	<0.0001			
**DUNNETT'S POST HOC TEST**				
	n	Mean Diff.	q	Summary
W	4			
E	4	2.1812	14.28	***
L1	4	0.3575	2.340	ns
L2	4	0.3237	2.119	ns
L3	4	0.0487	0.3190	ns
preS	4	-0.2575	1.686	ns
S	4	-0.5525	3.617	**

TERT-B (Figure 2B)

BROWN-FORSYTHE test				
Df:	6, 21			
F:	2.5720			
P value:	0.052			
**ONE-WAY ANOVA**				
Df:	6, 21			
F:	34.2769			
P value:	<0.0001			
**DUNNETT'S POST HOC TEST**				
	n	Mean Diff.	q	Summary
W	4			
E	4	1.7662	11.34	***
L1	4	0.4749	3.050	*
L2	4	0.5537	3.556	**
L3	4	0.3287	2.111	ns
preS	4	0.0125	0.08031	ns
S	4	-0.1363	0.8755	ns

TERT-B / TERT-A (Figure 2C)

BROWN-FORSYTHE test				
Df:	6, 21			
F:	0.9412			
P value:	0.4870			
**ONE-WAY ANOVA**				
Df:	6, 21			
F:	9.6250			
P value:	<0.0001			
**DUNNETT'S POST HOC TEST**				
	n	Mean Diff.	q	Summary
W	4			
E	4	-0.4150	3.331	*
L1	4	0.1175	0.9434	ns
L2	4	0.2300	1.846	ns
L3	4	0.2800	2.248	ns
preS	4	0.2700	2.168	ns
S	4	0.4163	3.342	*

TERT-A (Figure 2D)

BROWN-FORSYTHE test			
Df:	4, 14		
F:	0.4317		
P value:	0.7835		
**ONE-WAY ANOVA**			
Df:	4, 14		
F:	220.6		
P value:	<0.0001		
**POST HOC (Bonferroni mult. comp.)**			
	Mean Diff.	t	Summary
W / NQ	-0.08000	0.5851	ns
W / NK	-0.1225	0.8498	ns
NQ / NK	-0.04249	0.3107	ns
NQ / O	-3.420	22.97	***
NK / T	-2.361	15.16	***

TERT-B (Figure 2E)

BROWN-FORSYTHE test			
Df:	4, 14		
F:	2.916		
P value:	0.0601		
**ONE-WAY ANOVA**			
Df:	4, 14		
F:	5.304		
P value:	0.0082		
**POST HOC (Bonferroni mult. comp.)**			
	Mean Diff.	t	Summary
W / NQ	0.3581	3.278	*
W / NK	0.3887	3.376	*
NQ / NK	0.03062	0.2803	ns
NQ / O	-0.3033	2.551	ns
NK / T	-0.3449	2.774	ns

TERT-B / TERT-A (Figure 2F)

BROWN-FORSYTHE test			
Df:	4, 14		
F:	2.4171		
P value:	0.0978		
**ONE-WAY ANOVA**			
Df:	4, 14		
F:	43.14		
P value:	<0.0001		
**POST HOC (Bonferroni mult. comp.)**			
	Mean Diff.	t	Summary
W / NQ	1.058	5.41	***
W / NK	1.228	5.958	***
NQ / NK	0.1702	0.8698	ns
NQ / O	1.482	6.961	***
NK / T	1.068	4.797	**

Signif. codes: ***, $p < 0.001$; **, $p < 0.01$; *, $p < 0.05$

Experimental groups:

E ... egg
L1 - 3 ... larval stages
W … worker
preS ... presoldier
S ... soldier
NQ ... neotenic queen
NK ... neotenic king
O ... ovaria
T ... testes

Table S3b. Test statistics relative to the results presented in Figure 2G–L

TERT1 (Figure 2G)

BROWN-FORSYTHE test				
Df:	6, 21			
F:	0.4501			
P value:	0.8367			
**ONE-WAY ANOVA**				
Df:	6, 21			
F:	134.1			
P value:	<0.0001			
**DUNNETT'S POST HOC TEST**				
	n	Mean Diff.	q	Summary
W	4			
E	4	-3.836	23.10	***
L1	4	-0.7912	4.765	***
L2	4	-0.8587	5.172	***
L3	4	-0.4887	2.943	*
preS	4	-0.2025	1.220	ns
S	4	0.1013	0.6099	ns

TERT2 (Figure 2H)

BROWN-FORSYTHE test				
Df:	6, 21			
F:	1.375			
P value:	0.2702			
**ONE-WAY ANOVA**				
Df:	6, 21			
F:	15.68			
P value:	<0.0001			
**DUNNETT'S POST HOC TEST**				
	n	Mean Diff.	q	Summary
W	4			
E	4	-1.418	6.402	***
L1	4	-0.06250	0.2823	ns
L2	4	-0.1275	0.5759	ns
L3	4	0.2050	0.9258	ns
preS	4	0.5387	2.433	ns
S	4	0.1387	0.6263	ns

TERT1 / TERT2 (Figure 2I)

BROWN-FORSYTHE test				
Df:	6, 21			
F:	1.109			
P value:	0.3900			
**ONE-WAY ANOVA**				
Df:	6, 20			
F:	15.59			
P value:	<0.0001			
**DUNNETT'S POST HOC TEST**				
	n	Mean Diff.	q	Summary
W	4			
E	4	-0.4319	4.101	**
L1	4	-0.6584	6.252	***
L2	4	-0.6608	6.275	***
L3	4	-0.6191	5.878	***
preS	4	-0.6756	6.415	***
S	3	-0.06425	0.6101	ns

TERT1 (Figure 2J)

BROWN-FORSYTHE test			
Df:	4, 15		
F:	0.2404		
P value:	0.9110		
**ONE-WAY ANOVA**			
Df:	4, 15		
F:	32.55		
P value:	<0.0001		
**POST HOC (Bonferroni mult. comp.)**			
	Mean Diff.	t	Summary
W / NQ	-0.6825	2.460	ns
W / NK	-0.2975	1.221	ns
NQ / NK	0.3850	1.451	ns
NQ / O	-1.139	4.104	**
NK / T	-2.093	8.586	***

TERT2 (Figure 2K)

BROWN-FORSYTHE test			
Df:	4, 15		
F:	0.1430		
P value:	0.9633		
**ONE-WAY ANOVA**			
Df:	4, 15		
F:	99.38		
P value:	<0.0001		
**POST HOC (Bonferroni mult. comp.)**			
	Mean Diff.	t	Summary
W / NQ	0.08088	0.3499	ns
W / NK	0.4515	2.224	ns
NQ / NK	0.3706	1.677	ns
NQ / O	-2.966	12.83	***
NK / T	-2.516	12.39	***

TERT1 / TERT2 (Figure 2L)

BROWN-FORSYTHE test			
Df:	4, 15		
F:	0.4390		
P value:	0.9980		
**ONE-WAY ANOVA**			
Df:	4, 15		
F:	24.69		
P value:	<0.0001		
**POST HOC (Bonferroni mult. comp.)**			
	Mean Diff.	t	Summary
W / NQ	-0.7663	4.585	**
W / NK	-0.7202	4.907	***
NQ / NK	0.04601	0.2879	ns
NQ / O	1.318	7.887	***
NK / T	0.4358	2.969	*

Signif. codes: ***, $p < 0.001$; **, $p < 0.01$; *, $p < 0.05$

Experimental groups:

E ... egg
L1 - 3 ... larval stages
W … worker
preS ... presoldier
S ... soldier
NQ ... neotenic queen
NK ... neotenic king
O ... ovaria
T ... testes

Table S4. Test statistics relative to the results presented in Figure 3

ELISA somatic TERT (Figure 3A - extranuclear)

BROWN-FORSYTHE test				
Df:	4, 10			
F:	0.2587			
P value:	0.8977			
**ONE-WAY ANOVA**				
Df:	4, 10			
F:	7.039			
P value:	0.0058			
**DUNNETT'S POST HOC TEST**				
	n	Mean Diff.	q	Summary
W	3			
YQ	3	-1.170	3.675	*
YK	3	-1.474	4.630	**
MQ	3	-1.271	3.993	**
MK	3	-0.6227	1.956	ns

ELISA somatic TERT (Figure 3A - nuclear)

BROWN-FORSYTHE test				
Df:	4, 10			
F:	0.538			
P value:	0.712			
**ONE-WAY ANOVA**				
Df:	4, 10			
F:	1.645			
P value:	0.2383			
**DUNNETT'S POST HOC TEST**				
	n	Mean Diff.	q	Summary
W	3			
YQ	3	-0.003509	0.01248	ns
YK	3	0.4741	1.686	ns
MQ	3	0.3826	1.361	ns
MK	3	0.5108	1.817	ns

ELISA somatic TERT extranuclear/nuclear (Figure 3B)

BROWN-FORSYTHE test				
Df:	4, 10			
F:	0.2209			
P value:	0.9207			
**ONE-WAY ANOVA**				
Df:	4, 10			
F:	10.39			
P value:	0.0014			
**DUNNETT'S POST HOC TEST**				
	n	Mean Diff.	q	Summary
W	3			
YQ	3	-1.166	3.577	*
YK	3	-1.948	5.974	***
MQ	3	-1.654	5.072	**
MK	3	-1.133	3.476	*

ELISA TERT in cytoplasm, nucleus , mitochondria (Figure 3C)

cytoplasm		nucleus		mitochondria	
**F test for variances**		**F test for variances**		**F test for variances**	
Df:	2, 3	Df:	3, 2	Df:	2, 2
F:	3.355	F:	3.186	F:	8.165
P value:	0.3434	P value:	0.4982	P value:	0.2182
**t-test**		**t-test**		**t-test**	
Df:	5	Df:	5	Df:	4
t:	2.604	t:	0.2594	t:	0.117
P value:	0.0474	P value:	0.8057	P value:	0.9125

ELISA extranuclear TERT in gonads (Figure 3E)

BROWN-FORSYTHE test				
Df:	4, 11			
F:	0.7890			
P value:	0.5560			
**ONE-WAY ANOVA**				
Df:	4, 11			
F:	2.261			
P value:	0.1284			
**DUNNETT'S POST HOC TEST**				
	n	Mean Diff.	q	Summary
W	3			
YQ	3	0.1859	1.051	ns
YK	3	0.3825	2.163	ns
MQ	3	0.1937	1.171	ns
MK	3	0.4806	2.718	ns

Signif. codes: ***, $p < 0.001$; **, $p < 0.01$; *, $p < 0.05$

Experimental groups:

W ... worker
YQ ... young queen (<6 months)
MQ ... mature queen (>2 years)
YK ... young king (<6 months)
MK ... mature king (>2 years)

```
                                                                                         psTERT-altTSS_A-F
psTERT gene    1 AATTGCTAACGAAAACCAACAAAATGCACAAATGTTTCGGTCGTGCTTGACCATTTTCAGGGTGCGCTGTTACAGAGTAAGTGATACAACGACGTGTTCA 100
psTERT1          AATTGCTAACGAAAACCAACAAAATGCACAAATGTTTCGGTCGTGCTTGACCATTTTCAGGGTGCGCTGTTACAGA------------------------
                                                             M  F  R  S  C  L  T  I  F  R  V  R  C  Y  R  -  -  -  -  -  -  -  -
                                                                          putative signal peptide
psTERT2β         AATTGCTAACGAAAACCAACAAAATGCACAAATGTTTCGGTCGTGCTTGACCATTTTCAGGGTGCGCTGTTACAGAGTAAGTGATACAACGACGTGTTCA
psTERT2                                                                 TTCAGGGTGCGCTGTTACAGAGTAAGTGATACAACGACGTGTTCA

                         psTERT-TSS1-qPCR-F                      exon 1 ┐┌ exon 2
psTERT gene  101 TTCGTCCAACGTGTAATTGTTCAGACATATATTTCCACAATCCAAATATCGTGTGGCTGTGGTCTTCTGGGTTGTGACACTGTGTAGTCTTTTGACAGA 200
psTERT1          -------------------------------------------------------GCTGTGGTCTTCTGGGTTGTGACACTGTGTAGTCTTTTGACAGA
                  -  -  -  -  -  -  -  -  -  -  -  -  -  -  -  -  -  - A  V  V  F  W  V  V  T  L  C  S  L  L  T  D

psTERT2          TTCGTCCAACGTGTAATTGTTCAGACATATATTTCCACAATCCAAATATCGTGTGGCTGTGGTCTTCTGGGTTGTGACACTGTGTAGTCTTTTGACAGA

                                                  psTERT_EX2_R1 exon 2 ┐┌ exon 3
psTERT gene  201 CTGCTGTTGTTTTGAGAAAGCTACTCTATCCATCTTCAACCAAGTCTTGTGTTGGATGAACAAAGACATTTCTCACAAGCATATAAGAAGAAGAAAAAAG 300
psTERT1          CTGCTGTTGTTTTGAGAAAGCTACTCTATCCATCTTCAACCAAGTCTTGTGTTGGATGAACAAAGACATTTCTCACAAGCATATAAGAAGAAGAAAAAAG
                  C  C  C  F  E  K  A  T  L  S  I  F  N  Q  V  L  C  W  M  N  K  D  I  S  H  K  H  I  R  R  R  K  K

psTERT2          CTGCTGTTGTTTTGAGAAAGCTACTCTATCCATCTTCAACCAAGTCTTGTGTTGGATGAACAAAGACATTTCTCACAAGCATATAAGAAGAAGAAAAAAG
                                                                             M  N  K  D  I  S  H  K  H  I  R  R  R  K  K
                                                                                                      nuclear signal
                         psTERT-altTSS_A-R
psTERT gene  301 AAAATAAAGCCAAATGTCAGCACTGACAGCAGAATAGGATACAAGGGAACAACCGTATTGTCAAACAAACAAATGCTGTATAACCAAGCTACACATGAGC 400
psTERT1          AAAATAAAGCCAAATGTCAGCACTGACAGCAGAATAGGATACAAGGGAACAACCGTATTGTCAAACAAACAAATGCTGTATAACCAAGCTACACATGAGC
                  K  I  K  P  N  V  S  T  D  S  R  I  G  Y  K  G  T  T  V  L  S  N  K  Q  M  L  Y  N  Q  A  T  H  E

psTERT2          AAAATAAAGCCAAATGTCAGCACTGACAGCAGAATAGGATACAAGGGAACAACCGTATTGTCAAACAAACAAATGCTGTATAACCAAGCTACACATGAGC
                  K  I  K  P  N  V  S  T  D  S  R  I  G  Y  K  G  T  T  V  L  S  N  K  Q  M  L  Y  N  Q  A  T  H  E

                                                        ...

                                  psTERT-TES-qPCR-F2                          exon 8 ┐┌ exon 9
psTERT gene 2001 TGATTATGCTCGCTACAGGTTTCAAGATATTGCCCATTCCATGCGTCTGACAGAAGTGAAATGTCCTGGAAAGTACCTTCAGAAGCGGATGGAAGGTTCA 2100
psTERT-A,B       TGATTATGCTCGCTACAGGTTTCAAGATATTGCCCATTCCATGCGTCTGACAGAAGTGAAATGTCCTGGAAAGTACCTTCAGAAGCGGATGGAAGGTTCA
                  D  Y  A  R  Y  R  F  Q  D  I  A  H  S  M  R  L  T  E  V  K  C  P  G  K  Y  L  Q  K  R  M  E  G  S

psTERT gene 2101 TTTTCTTCATTAAAACTGAATGTTCTTGTCCTGGATGAGCTGTACAACTCAAAAGATGTAGTTCTCACAAACGTATTTTATGCAGGTCTTTTGCAGGCTT 2200
psTERT-A,B       TTTTCTTCATTAAAACTGAATGTTCTTGTCCTGGATGAGCTGTACAACTCAAAAGATGTAGTTCTCACAAACGTATTTTATGCAGGTCTTTTGCAGGCTT
                 F  S  S  L  K  L  N  V  L  V  L  D  E  L  Y  N  S  K  D  V  V  L  T  N  V  F  Y  A  G  L  L  Q  A  F

psTERT gene 2201 TCAGATTCCACAGTCTTGTTAAATGTACCTTTGATAAGAAGAATCTGAATCAGCAGTTTCTTACCCAAATAGTGTATGAAACTGTCGGTAGGACTGGGGG 2300
psTERT-A,B       TCAGATTCCACAGTCTTGTTAAATGTACCTTTGATAAGAAGAATCTGAATCAGCAGTTTCTTACCCAAATAGTGTATGAAACTGTCGGTAGGACTGGGGG
                 R  F  H  S  L  V  K  C  T  F  D  K  K  N  L  N  Q  Q  F  L  T  Q  I  V  Y  E  T  V  G  R  T  G  G

                                                                 psTERT-TES1-qPCR-R          exon 9 ┐┌ exon 10
psTERT gene 2301 ACACACATTGAATATTGCCAAGAAATACTGCAAAGGTAATGCGCCAAGAAAATATAGAGACAGAATAAACCTCTATTAATCCACTTCGACGCTTGACTTA 2400
psTERT-B         ACACACATTGAATATTGCCAAGAAATACTGCAAAGGTAATGCGCCAAGAAAATATAGAGACAGAATAAACCTCTATTAATCCA
                  H  T  L  N  I  A  K  K  Y  C  K  G  N  A  P  R  K  Y  R  D  R  I  N  L  Y  *

psTERT-A         ACACACATTGAATATTGCCAAGAAATACTGCAAAG------------------------------------------------CTTCGACGCTTGACTTA
                  H  T  L  N  I  A  K  K  Y  C  K  -  -  -  -  -  -  -  -  -  -  -  -  -  -  -  -  A  S  T  L  D  L
                    psTERT-TES2-qPCR-R3
psTERT gene 2401 AATGCTGTTCGCTGGATGATGTACAGAGCTTTCTTCTTGAGATTGTCCATTGTTCAAAGCTACTATCGTCTTGTACTGAGAAACATTGATTGGATGTTAA 2500
psTERT-A         AATGCTGTTCGCTGGATGATGTACAGAGCTTTCTTCTTGAGATTGTCCATTGTTCAAAGCTACTATCGTCTTGTACTGAGAAACATTGATTGGATGTTAA
                  N  A  V  R  W  M  M  Y  R  A  F  F  L  R  L  S  I  V  Q  S  Y  Y  R  L  V  L  R  N  I  D  W  M  L
                                               conserved region E-III

psTERT gene 2501 AGCGTCTTTCTGAAAGGATACCAGATGAAAAATGTGATCAGATGATGCAGCTAACGTTACGCATTCCAGATGCATTTAAAACAGTTTCATAAAATGTTGC 2600
psTERT-A         AGCGTCTTTCTGAAAGGATACCAGATGAAAAATGTGATCAGATGATGCAGCTAACGTTACGCATTCCAGATGCATTTAAAACAGTTTCATAAAATGTTGC
                 K  R  L  S  E  R  I  P  D  E  K  C  D  Q  M  M  Q  L  T  L  R  I  P  D  A  F  K  T  V  S  *
                                                                 conserved region E-IV

psTERT gene 2601 CGCGCTTCTGCATGAACATGTTAGGTACTGGTGTGCCATGTGACACAGTCTTTAGGCACTTAACTTTGATTGCTTAATATGAACAAATAGTTGGCTAGAT 2700
psTERT-A         CGCGCTTCTGCATGAACATGTTAGGTACTGGTGTGCCATGTGACACAGTCTTTAGGCACTTAACTTTGATTGCTTAATATGAACAAATAGTTGGCTAGAT

psTERT gene 2701 GACCAAGCC                                                                                            2709
psTERT-A         GACCAAGCC
```

Figure S2. Nucleotide and protein sequence alignment representing alternative splicing in exons 1 and 9 and in isoforms TERT1, 2 and TERT-A, B. Genomic reference is in bold and shaded in gray, primer annealing sites used for isoform-specific expression analyses are underlined and shown in blue (forward) and green (reverse). Functionally important regions such as putative signal peptide (pink), nuclear signal (orange) and C-terminal conserved regions EIII and EIV (both blue) are shaded and bold-faced. Numbers represent positions in full transcript sequence containing all *psTERT* exons, exon junctions are represented by broken lines.

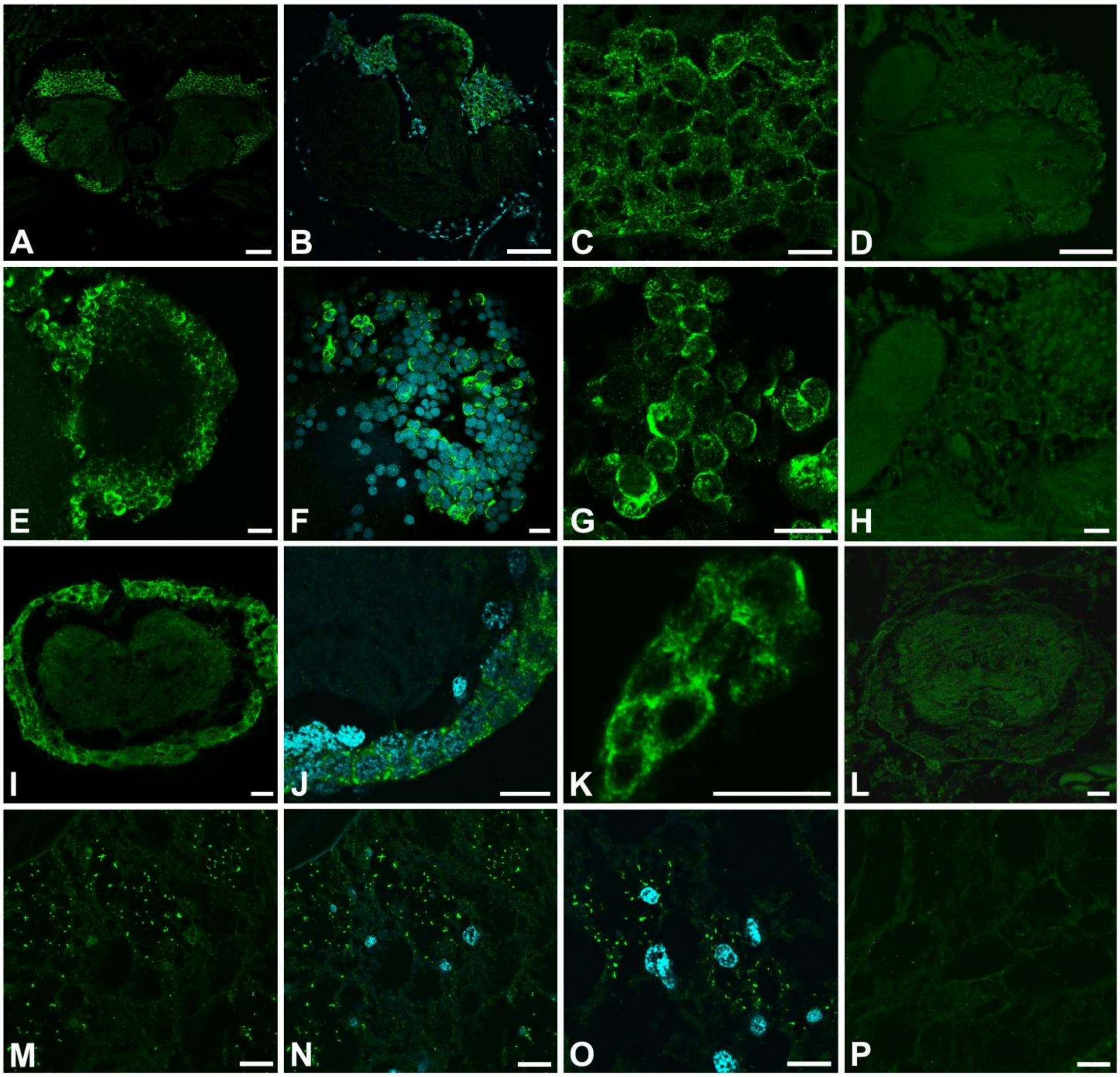

Figure S3. Immunodetection of psTERT in the central nervous system and in the fat body of *P. simplex* workers. **A-C.** Paraplast sections with psTERT positive staining in brain cells. **D.** Absence of positive signal in the control brain section when primary antibody was replaced by preimmune serum. **E-G.** Immunodetection of psTERT in wholemount preparations of the brain revealed an identical staining pattern as on paraplast sections. The images show different magnifications of the optic lobe region. **H.** High magnification image of the control sample shown in D. **I-K.** Different magnifications of psTERT immunoreactivity in paraplast section of an abdominal ganglion of the ventral nerve cord. **L.** Absence of positive signal in a ganglion of the control sample. **M-O.** Different magnifications of psTERT immunoreactivity in paraplast sections of fat body. **P.** Absence of positive signal in the control fat body sample. In B, F, J, N and O, the psTERT signal (green) is merged with DAPI staining (blue). Scale bars represent 50 µm in A, B, D and 10 µm in C, E-P.

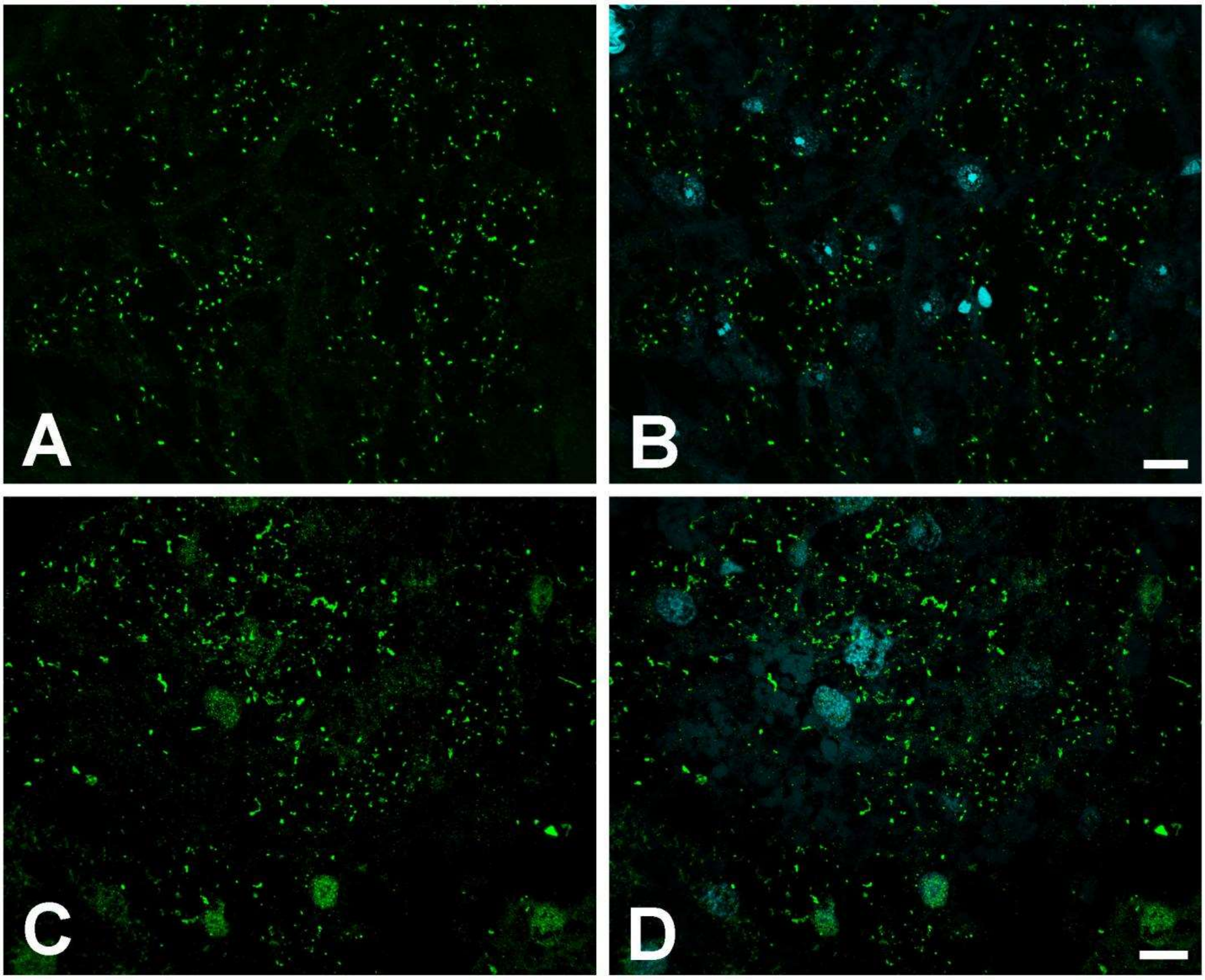

Figure S4. Abundant psTERT-RP immunoreactivity in fat body of some queens and kings. A, B. Fat body of queen. **C, D.** Fat body of king. Left - labelling with anti-psTERT-RP antibody, right - psTERT-RP signal (green) merged with DAPI staining (blue). Scale bar = 10 μm.

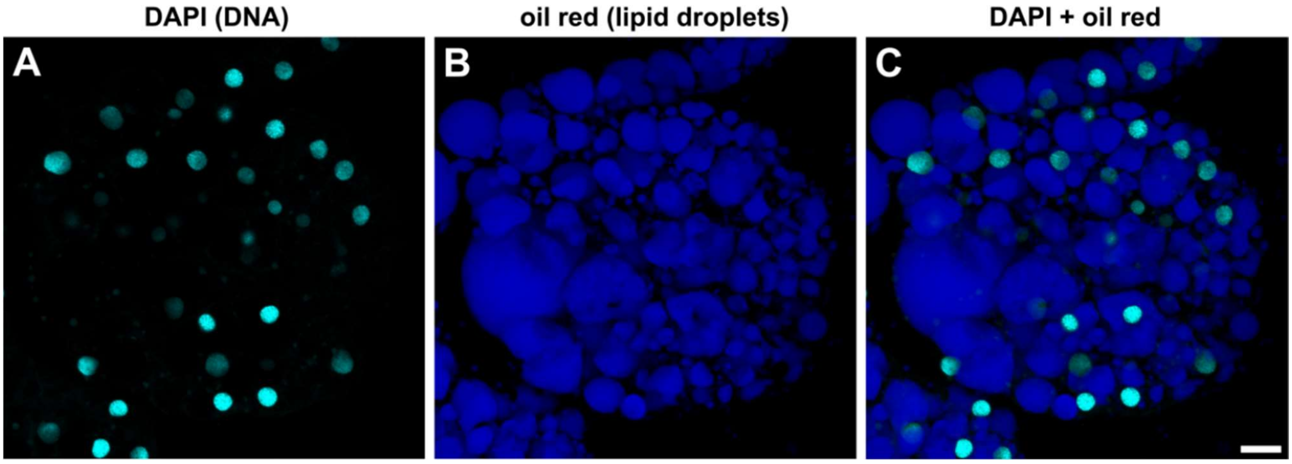

Figure S5. Structure of adipous tissue of the fat body in workers. Paraplast section of the fat body stained with DAPI (A), oil red (B) and DAPI + oil red (C) to visualize lipid droplets and nuclei in adipocytes. The extranuclear cytoplasm is marginalized by the large lipid droplets, filling the majority of the cell volume. Scale bar represents 15 µm.

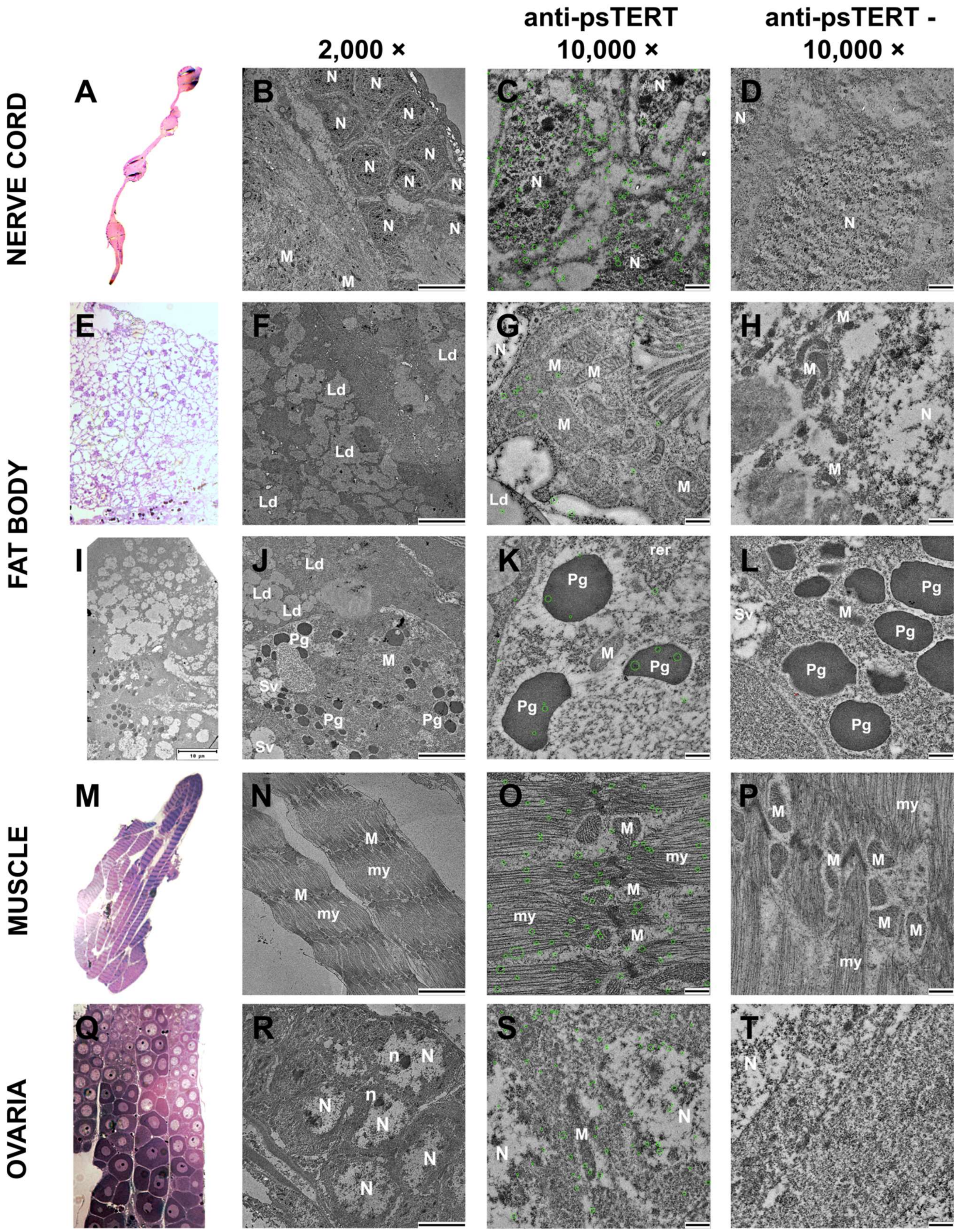
2,000 ×
anti-psTERT 10,000 ×
anti-psTERT - 10,000 ×
NERVE CORD
FAT BODY
MUSCLE
OVARIA
A
B
C
D
E
F
G
H
I
J
K
L
M
N
O
P
Q
R
S
T
N
M
Ld
Pg
Sv
rer
my
n

Figure S6. Transmission electron microscopy (TEM) with psTERT detection using anti-psTERT antibody and immunogold labeling. Immunoreactions were performed on ultrathin sections of tissue samples dissected from *P. simplex* neotenic queens: ventral nerve cord (A-B), abdominal fat body (E-L), muscle (M-P) and ovaria (Q-T). **(A, E, M, Q)**. Semithin sections of examined tissues stained with toluidine blue and taken under optical microscope. **I.** TEM micrograph at 1000x magnification showing multiple cell types in the fat body tissue. **(B,F,J,N,R)**. TEM micrographs at 2000x magnification showing structure of examined tissues, scale bar 5 μm. **(C,G,K,O,S)**. TEM micrographs at 10000x magnification with immunochemical detection of psTERT, scale bar 500 nm. Cellular organelles are marked with white symbols: N - nucleus, n - nucleolus, M -mitochondria, Ld - lipid droplets, Sv - secretory vesicles, Pg -protein granules, rer - rough endoplasmic reticulum, my - myofibrils.

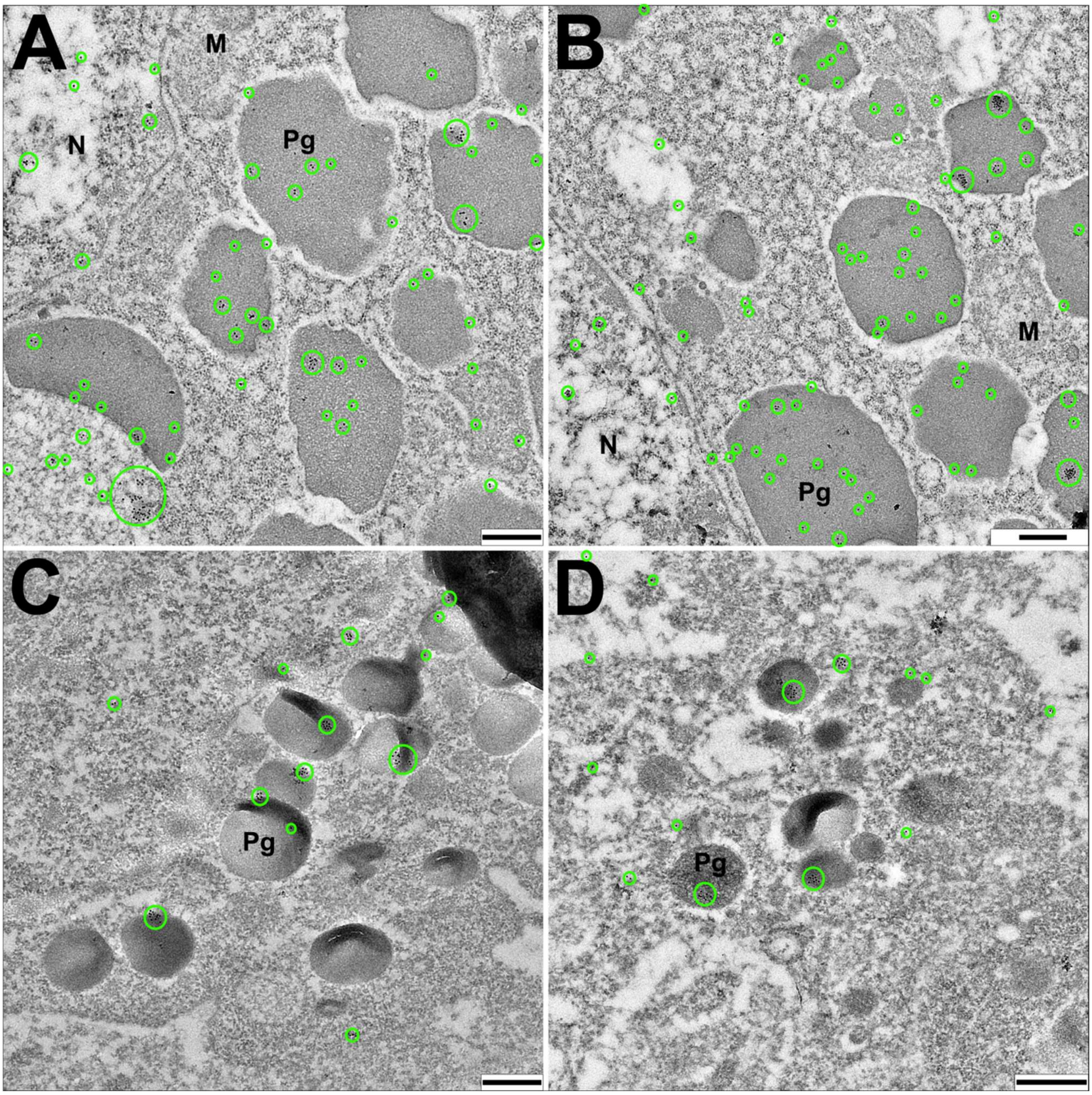

Figure S7. Transmission electron microscopy (TEM) with immunogold detection showing psTERT accumulation to two types of protein granules on ultrathin sections of fat body from *P. simplex* neotenic queens. (A, B). Typical proptein granules from adipous tissue of *P. simplex* abdominal fat body. **(C, D)**. Protein granules on fat body sections found commonly in the proximity of other organs such as reproductive organs or ventral nerve cord. Cellular organelles are marked by black symbols: N - nucleus, M - mitochondria, Pg - protein granules. Scale bar represents 500 nm.

Table S5. Most frequent splice events observed in hTERT according to Ludlow *et al.* (2019).

isoform	Splicing event	Termination codon	RT activity	Function
**full-length**	none	Standard, exon 16	full	Telomere maintenance
**DEL2**	Deletion of exon 2	PTC, exon 3	No	Mostly degraded
**minus-alpha**	Partial deletion of exon 6	Standard, exon 16	No	Dominant-negative, binds *hTERC*
**minus-beta**	Deletion of exons 7, 8	PTC, exon 10	No	Mostly degraded, may play a role in DNA repair
**minus gamma**	Deletion of exon 11	Standard, exon 16	No	Dominant-negative, binds hTERC, tissue-specific
**delta4-13**	Deletion of exons 4-13	Standard, exon 16	No	Proposed to stimulate proliferation
**INS3**	Partial insertion of intron 14	PTC, intron 14	partial	Dominant-negative, binds *hTERC*, tissue-specific
**INS4**	Partial insertion of intron 14	PTC, exon 14	partial	Dominant-negative, binds *hTERC*, tissue-specific

Legend: PTC - preliminary termination codon, hTERC – human telomerase RNA component

Supplementary reference:

Ludlow, A.T., Slusher, A.L., Sayed, M.E. (2019) Insights into telomerase/hTERT alternative splicing regulation using bioinformatics and network analysis in cancer. *Cancers* 11: 666.
